# X-ray Micro-Computed Tomography: A Powerful Device to Analyze the 3D Microstructure of Anode-Electrolyte in BaZr_0.8_Y_0.2_O_3_ Protonic Ceramic Electrochemical Cells and the Reduction Behavior

**DOI:** 10.3390/membranes12010068

**Published:** 2022-01-04

**Authors:** Victoire Lescure, Morgane Gelin, Mélanie François, Mohammad Arab Pour Yazdi, Pascal Briois, Frédéric Demoisson, Lionel Combemale, Solène Valton, Gilles Caboche

**Affiliations:** 1 Laboratoire Interdisciplinaire Carnot de Bourgogne, FCLAB, ICB-UMR6303, CNRS, Université de Bourgogne Franche-Comté, 9 Avenue Savary, BP47870, CEDEX, 21078 Dijon, France; victoire_lescure@etu.u-bourgogne.fr (V.L.); melanie.francois@u-bourgogne.fr (M.F.); frederic.demoisson@u-bourgogne.fr (F.D.); lionel.combemale@u-bourgogne.fr (L.C.); 2 Institut FEMTO-ST, FCLAB, UMR 6174, CNRS, University Bourgogne Franche-Comté, 15B Avenue des Montboucons, 25030 Besançon, France; mohammad.arab-pour-yazdi@utbm.fr (M.A.P.Y.); pascal.briois@utbm.fr (P.B.); 3 RX Solutions SAS, 24 Bis Rue Uranus, ZAC Altaïs, 74650 Chavanod, France; morgane.gelin@rx-solutions.com (M.G.); solene.valton@rx-solutions.com (S.V.)

**Keywords:** protonic ceramic fuel cell, X-ray micro-computed tomography, BaZr_0.8_Y_0.2_O_3−δ_, reactive pulsed DC magnetron sputtering

## Abstract

New advanced fuel cell technologies are moving towards high-temperature proton conductors (HTPCs) to meet environmental issues. Their elaboration remains a challenge and micro-computed tomography (µCT) is an innovative way to control their quality. NiO-BZY anodic supports of a protonic ceramic electrochemical cell (PCEC), elaborated by co-tape casting and co-sintered at 1350 °C, were coated with a BZY20 electrolyte layer by DC magnetron sputtering. The µCT allowed to observe defects inside the volume of these PCEC half-cells and to show their evolution after an annealing treatment at 1000 °C and reduction under hydrogen. This technique consists in obtaining a 3D reconstruction of all the cross-sectional images of the whole sample, slice by slice. This allows seeing inside the sample at any desired depth. The resolution of 0.35 µm is perfectly adapted to this type of problem considering the thickness of the different layers of the sample and the size of the defects. Defects were detected, and their interpretation was possible thanks to the 3D view, such as the phenomenon of NiO grain enlargement explaining defects in the electrolyte, the effect of NiO reduction, and finally, some anomalies due to the shaping process. Ways to anticipate these defects were then proposed.

## 1. Introduction

Protonic ceramic electrical cells (PCEC) have an increasing research activity and show greater abilities for high performances and reversibility [1,2,3]. The use of high-temperature proton conductors (HTPCs) in such a system decreases the operating temperature in the 400–600 °C range and promotes better durability of the cells [1,2,4].

Among the perovskite material HTPCs, Y-doped BaCeO_3_ exhibits the highest total conductivity at 600 °C [5,6,7]. However, its poor stability against CO_2_ and H_2_O-containing atmosphere hinder its practical application [8,9,10,11]. In contrast, Y-doped BaZrO_3_, also called BZY, shows high chemical stability and an acceptable total conductivity at 600 °C, typically 10^−3^ S·cm^−1^ [12,13,14,15,16]. For these reasons, BZY is widely considered a promising electrolyte candidate for PCEC [17,18,19]. Furthermore, high electrochemical performances are guaranteed as the electrolyte gets smaller and denser.

The major limitation of BZY is its grain boundary resistance that limits its total conductivity [20,21,22]. Moreover, the highly refractive nature of BZY requires high sintering temperatures (1600–1700 °C) and long annealing times (>24 h), to achieve dense membranes with large grains and, hence, increase its performances [23,24]. 

Recently, the elaboration of thin and highly textured BZY electrolyte by pulsed laser deposition or reactive DC sputtering has been developed in order to overcome the grain boundary resistance drawback [25,26,27]. However, both techniques are highly sensitive to the substrate surface quality (flatness, roughness and densification) [28,29]. To this extent, having more information on the quality of the microstructure of the substrate and the electrolyte and the morphology of the interface between the two layers is important. 

In this work, we used micro X-ray tomography, a powerful characterization device allowing a tridimensional morphological analysis, to detect defects in PCEC half-cells that could lead to sample failure during operation. Other more classical characterization methods could be used, such as scanning electron microscopy (SEM) [30] or transmission electron microscopy (TEM) [31]. However, these methods only allow a single plan view at a time, whereas µCT is a way to visualize every single plan view of the sample. The substrate consisting of a porous and thick anode, and a thinner and denser layer on top called anode functional layer (AFL), is elaborated by co-tape casting, both made by the widely used cermet Ni-BaZr_0.8_Y_0.2_O_3−δ_ (noted BZY20) [32,33]. The anode-AFL assembly is co-sintered at 1350 °C for 5 h in the air to ensure the required mechanical properties and smooth surface [34,35]. The BZY20 electrolyte is deposited by reactive pulsed DC magnetron sputtering on top of the AFL layer for a final thickness of 2.5 µm. 

The X-ray micro-computed tomography (µCT) used in this study has a 0.35 µm resolution allowing the detection of small defects inputted during the elaboration process, especially in the bulk of anode and AFL and the interface AFL–electrolyte. Secondly, tomography was carried out to study the reduction of the anode and AFL layers. Three types of samples are studied: the raw half-cell, right after the deposition of the electrolyte on the anode with no added treatment, the annealed at 1000 °C half-cell and the reduced half-cell.

## 2. Materials and Methods

### 2.1. Materials

The starting powders of the substrate were provided by fuel cell materials (FCM, Cincinnati, OH, USA) for NiO (Ref: NiO-P, Lot#R5739, Item#312010, surface area 3.4 m^2^·g^−1^) and by Cerpotech (Cerpotech, Norway) for BZY20 (Lot#180120A, surface area 24 m^2^·g^−1^). The anode-AFL assembly was prepared by co-tape casting as described elsewhere [36]. The NiO-SDC powder was replaced by NiO and BZY20 powders in a 60/40 weight ratio. The substrates are cut with a 28 mm in diameter punch prior to the co-sintering. The heating and cooling rate are set at 3 °C·min^−1^, an isothermal plateau at 300 °C for 2 h is applied to eliminate the organics before the staying time of 5 h at 1350 °C. The final diameter of the substrates is 22 mm.

Then, the BZY electrolyte layer was deposited by co-sputtering of Ba (purity 99.9%, Ø 50 mm × 3 mm) and Zr_0.8_Y_0.2_ (purity 99.9%, Ø 50 mm × 6 mm) targets. The reactor was a 90 L cylinder Alcatel 604 SCM (CIT Alcatel, Annecy, France) pumped down via a turbo molecular pump system that permitted a residual vacuum below 10^−4^ Pa. The chamber was equipped with circular planar and water-cooled magnetron sputtering sources and the rotating substrate holder was parallel to these sources at about 60 mm. The Ba and Zr_0.8_Y_0.2_ targets were supplied with a pulsed DC (Direct Current) advanced energy dual generator authorizing the control of the discharge power. 

Argon (50 mL·min^−1^) and oxygen (5 mL·min^−1^) flow rates were controlled with the Brooks flowmeters and the working pressure was kept at 1 Pa during deposition (working pressure measured using an MKS Baratron gauge). The anode-AFL substrates were placed on the rotating substrate holder at 40 mm from the substrate holder axis and were heated by radiative effect with a graphite electrical resistance heater placed behind the substrate-holder at 400 °C during deposition. The parameters of each target are presented in Table 1. The time of deposition was set to 1 h to obtain a layer of 2.5 µm.

Then, the half-cell undergoes an annealing treatment of 1000 °C for 2 h, with a heating rate of 3 °C·min^−1^ to ensure good densification. To study the effect of the reduction on the material, the sample is put under dry hydrogen gas with a flow of 66.7 mL·min^−1^. The reduction was carried out at 550 °C for 2 h, with a heating rate of 5 °C·min^−1^.

### 2.2. Characterization of the Samples

#### 2.2.1. Microstructure and Composition

The thickness and microstructure of the coating were measured and observed on a brittle fracture cross-section and the top surface using a Hitachi SU8230 Scanning Electron Microscope (SEM). The chemical composition was verified by energy dispersive spectrometry (EDS) with a Thermo-Scientific Ultradry Detector.

#### 2.2.2. X-ray Tomography Analysis

X-ray tomography allows having not just a cross-section but information about the whole volume of the sample. It allows the 3D reconstruction from cross-sectional images and therefore allows the possibility to analyze the whole volume of the sample and visualize the cross-section at any desired depth in any direction. 

X-ray tomography first consists in acquiring a series of X-ray radiographs of a sample that rotates around an axis perpendicular to the incident beam. Each radiograph acquired during a scan is composed of pixels (here 1840 × 1456). Each pixel has a gray value which depends on the material density crossed at its position. The different gray values composing an image are then distributed in a histogram. 

The spatial X-ray attenuation distribution is then reconstructed by using a filtered back-projection algorithm, represented as a 3D volume. Detailed analysis of the half-cell morphology before and after annealing in air and in the reductive atmosphere are performed using an Easytom XL Ultra tomograph from the company RX Solutions LTD. A very high resolution (compared to currently available cone-beam laboratory systems) is achieved by using a Hamamatsu X-ray source. It achieves a spot size of 0.80 µm when using a W filament and it can even be reduced to 0.35 µm with a LaB_6_ cathode. The X-ray source was operated with a LaB_6_ cathode at a voltage of 100 kV. The detector is a Varex Flat Panel with a pixel size of 127 µm. The samples are scanned at a voxel size of 0.35 µm. Each scan consisted of around 1440 projections with an exposure time of 1 s and an averaging of 20 radiographs for each.

The tomography is based on the Beer–Lambert law of attenuation (Equation (1)):

(1) $$I=I_{0}e^{-µs}$$

where I is the measured intensity of the X-ray, I_0_ is the initial intensity of the X-ray, µ is the X-ray attenuation coefficient, and s is the length of sample crossed by the X-ray.

The attenuation coefficient of the Beer–Lambert law, which is used in X-ray radiography physics, is proportional to the fourth power of the atomic number (Z^4^) and the density ρ of the investigated material. The contrast observed in the X-ray radiograph is explained by this attenuation law.

### 2.3. Volume Reconstruction and Microstructure Characterization

The asset of X-ray µCT analysis is based on the information obtained: a 3D representation of the attenuation of the different elements composing the sample. 

**Figure 1:**
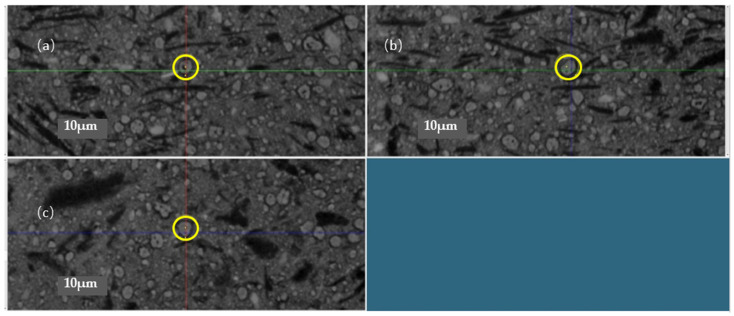
Grayscale representation of X-ray µCT slices of a sample in three different orientations: X (**a**), Y (**b**), Z (**c**). Identification and localization of a Ni grain (yellow circles) in the reconstructed volume.

Three cross-section slices, composed of voxels of different gray levels, allow characterizing nondestructively the microstructure of the sample scanned. Figure 1 shows an example of the three cross-sectionnal slices in X (a), Y (b) and Z (c) directions.

The different gray levels offer possibilities to segment elements of different densities and/or thicknesses, like inclusions, grains, pores, or even layers of different materials which can be randomly distributed in the sample. These elements can then be measured and or localized in 3D. 

**Figure 2:**
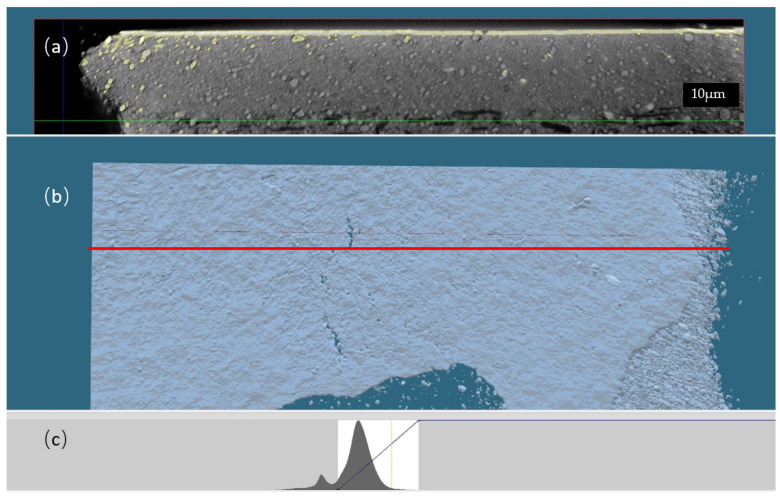
(**a**) Reconstructed slice localized inside the volume; its location is represented by the red line on figure (**b**). The 3D surface rendering (**b**) of a selected gray level, with the threshold represented by the yellow cursor on the figure (**c**). (**c**) Gray-level histogram of the tomograph.

**Figure 3:**
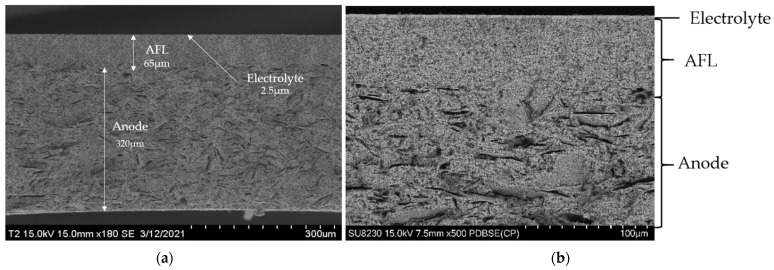
SEM micrographs of the cross-section of a sample with the three different layers. (**a**) general view in secondary electron mode, (**b**) detailed view in backscattering electron mode.

Moreover, X-ray µCT allows obtaining a 3D surface rendering of a specific gray level of the tomograph (Figure 2). A slice inside the volume with a specific orientation, X for example, can be chosen (Figure 2a), localized and presented in gray level (Figure 2c). Then, the surface corresponding to the selected gray level can be reconstructed in order to give a 3D surface rendering of the sample (Figure 3a).

## 3. Results and Discussion

### 3.1. Scanning Electron Microscopy and Elemental Content Analysis

SEM micrographs of the cross-section of a sample are shown in Figure 3. The microstructure of a typical half-cell is visible on image a. Each layer, anode, AFL and electrolyte, presents a homogeneous thickness, is highly covering and perfectly adherent to each other. The anode is 320 µm thick with the presence of lamellar porosity caused by carbon graphite pore former included during the elaboration process. The 65 µm-thick AFL is a lot denser. The electrolyte is 2.5 µm thick. 

**Figure 4:**
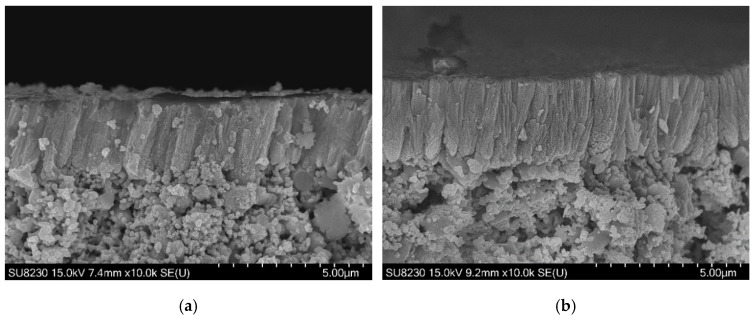
SEM micrographs of the electrolyte layer: (**a**) on the raw sample; (**b**) annealed sample at 1000 °C for 2 h.

The microstructure of the BZY20 layer as-deposited is shown in Figure 4a. The layer is dense, and no delamination was observed. Furthermore, grains are highly oriented, presenting the characteristic columnar shape of thin film deposited by physical vapor methods. This morphology is beneficial for the application as it eases the path of protons [27]. The columns are tilted due to the vapor flux angle [37,38]. Indeed, the Ba and Zr_0.8_Y_0.2_ targets were tilted by 15° to the normal to the surface of the support to ensure the best yield and homogeneity deposition and take into account the size of the targets.

The micrograph post-annealing is exposed in Figure 4b. The same dense columnar microstructure is observed, and the AFL–electrolyte interface is not altered by the thermal treatment. Furthermore, a straightening of the columns occurred. This reorientation could be attributed to stress relaxation [39,40].

The chemical composition was analyzed by Energy Dispersive Spectrometry (EDS) throughout the whole sample, meaning the three different layers, and the results are consistent with the expected BaZr_0.8_Y_0.2_O_3−δ_ composition.

### 3.2. X-ray Microtomography Characterization

**Figure 5:**
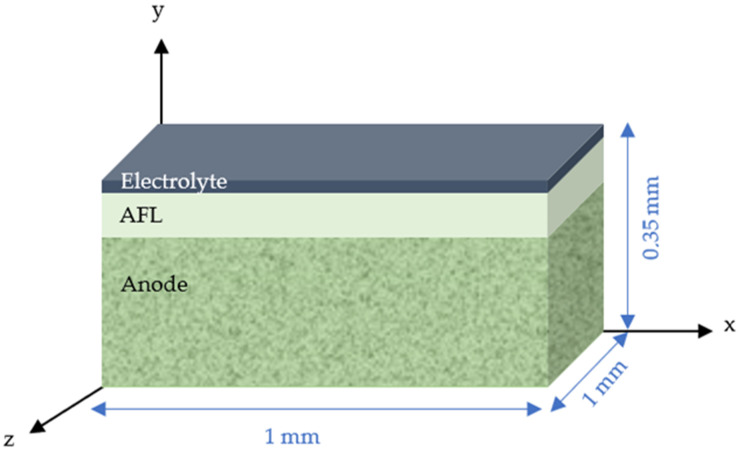
Schematic of the studied sample.

The aim is to study and interpret the origin of the defect due to the elaboration process that could lead to a decrease in cell performances. To be studied, a small piece of 1 × 1 × 0.38 mm^3^ was selected after breaking up for characterization by X-ray µCT. The prepared sample piece was successively analyzed first in the raw deposition state, then after annealing in air at 1000 °C for 2 h and finally after reduction in H_2_ at 550 °C for 2 h to understand the impact of these different treatments. Figure 5 shows the schematic of the studied samples, the axes direction used in the following paragraphs are also represented. 

**Figure 6:**
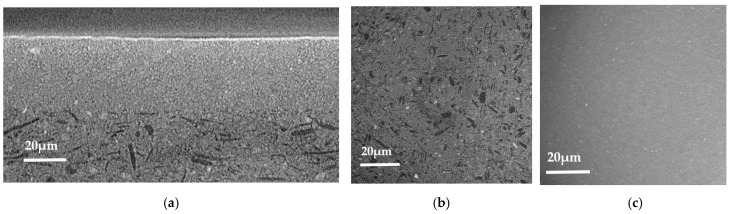
X-ray tomography images of the raw sample: (**a**) cross-section along the *X*-axis; (**b**) cross-section of the anode along the *Y*-axis; (**c**) cross-section of the AFL along the *Y*-axis.

The first sample to be studied is the raw BZY20 half-cell. Figure 6 exhibits typical cross-sectional tomographs of the raw BZY20 half-cell along the *X*-axis (Figure 6a), along the *Y*-axis in the anode layer (Figure 6b), and along the *Y*-axis in the AFL (Figure 6c). Since not only the nature of the material but also its density affects the attenuation coefficient in the Beer–Lambert law, the tomography allows obtaining an image in contrast to density. Then, in the tomographs, the porosity is in dark gray. BZY appears in white and Ni in light gray. The median gray corresponds to a mix of BZY and NiO; these two phases are not properly separated because the resolution of the tomograph (350 nm) is lower than the mean grain size of BZY (173 nm, evaluation of 50 particles on SEM micrographs). In Figure 6a, where all three layers are displayed, the same microstructure as in SEM is highlighted, i.e., the anode is porous, the AFL is denser, and the electrolyte is dense. This microstructure is the same in any direction, as shown by the cross-section along the *Y*-axis.

**Figure 7:**
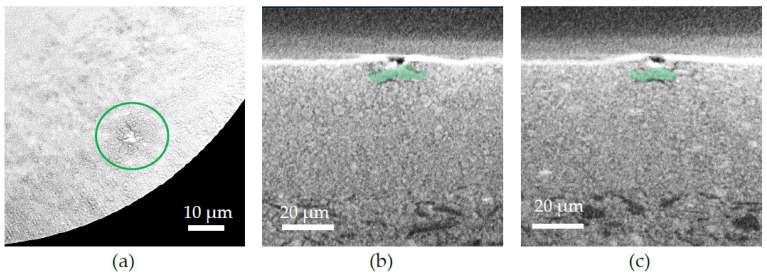
X-ray tomography images of a defect in the upper layers of the raw sample: (**a**) image from the top (*Y*-axis) of the defect in the electrolyte; (**b**) cross-section where the defect is along the *X*-axis with a NiO grain (in green) in the AFL; (**c**) cross-section where the defect is along the *Z*-axis with a NiO grain (in green) in the AFL.

Traveling through the sample along the *Y*-direction highlights an irregularity in the electrolyte layer present in the electrolyte (Figure 7a). Figure 7b,c show the same defect along the *X*- and *Z*-axis, respectively. A NiO grain bigger than usual highlighted in green on Figure 7 (10 µm compared to a mean size of 2–3 µm) is evidenced very close to the interface between the AFL and the electrolyte and is undoubtedly the cause of the crack of the electrolyte.

According to the tomography analysis, the defect in the electrolyte layer is attributed to the dramatic grain growth of a NiO grain in the AFL layer near the AFL–electrolyte interface. It has been reported that NiO grain growth is significantly accelerated at 850 °C [41]. Thus, during the sintering stage at 1350 °C, a grain coarsening and agglomeration occurred, leading to unusually large NiO grains.

Since the AFL thickness is 65 µm and the coarse NiO grains are 10 µm, the probability of having a big NiO grain near the electrolyte interface is relativity high (about 16%). Other coarse NiO grains were found within the AFL layer; only the grains near the electrolyte interface are detrimental.

The TEC (Thermal Expansion Coefficient) is 40% to 50% higher for the oxide nickel than for the BZY (12–14 × 10^−6^·K^−1^ for NiO [42] and 8–10 × 10^−6^·K^−1^ for BZY [43]).

Assuming the fact that, at this stage, there are unusually large NiO grains at the interface and that there is a differential expansion between the NiO grains and the BZY, the crack is formed during the cooling after the deposition at 450 °C. The heat during the deposition process caused the grain to expend more than the BZY matrix but also to shrink more afterward, dragging the electrolyte layer with it leading to a crack.

Furthermore, it has been reported that the NiO agglomeration is more susceptible to lead to a NiO grain growth [41], then increasing the grinding of the powder prior to the slurry preparation could decrease the mean grain size and decrease the probability of having coarse grain near to the AFL–electrolyte interface.

**Figure 8:**
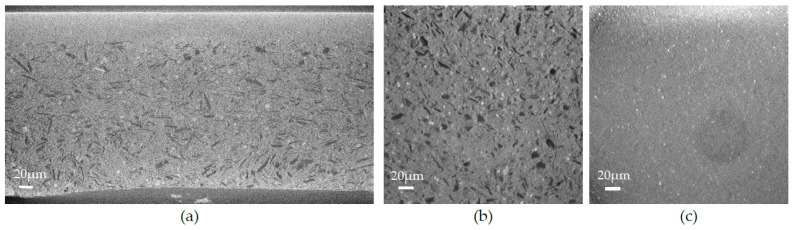
X-ray tomography images of the annealed sample: (**a**) cross-section along the *X*-axis; (**b**) cross-section of the anode along the *Y*-axis; (**c**) cross-section of the AFL along the *Y*-axis.

The next sample to be studied is the annealed half-cell. Figure 8a is an X-ray tomograph along the *X*-axis showing the cross-section of the fired half-cell where all three layers, anode, AFL, and electrolyte, are visible. Figure 8b,c are X-ray tomography images along the *Y*-axis showing the anode (porous) and the AFL (dense). The microstructure of the three layers is very similar to the raw half-cell exhibited in Figure 6, attesting that the annealing treatment does not alter the microstructure.

**Figure 9:**
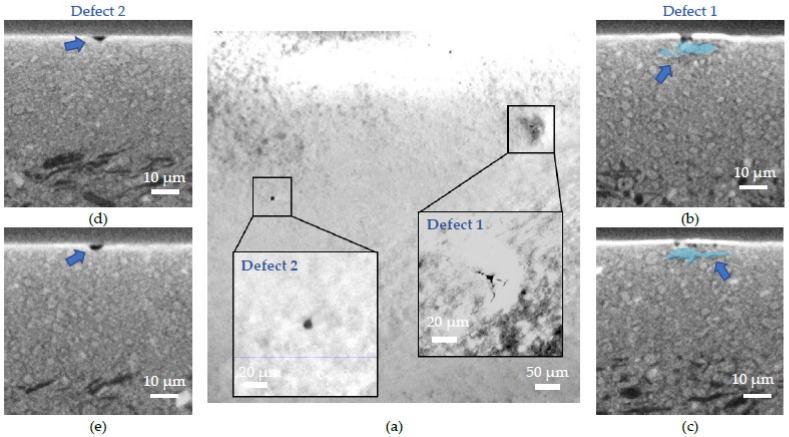
X-ray tomography images of defects in the annealed sample: (**a**) image along the *Y*-axis with focus on two defects, the inserts show higher magnification images of the defects; (**b**) cross-section of Defect 1 along the *X*-axis; (**c**) cross-section of Defect 1 along the *Z*-axis; (**d**) cross-section of Defect 2 along the *X*-axis; (**e**) cross-section of Defect 2 along *Z*-axis.

As previously, defects were highlighted thanks to the tomography analysis. Figure 9a shows the tomographs in the electrolyte layer along the *Y*-axis, Figure 9b,d are cross-section images along the *X*-axis and Figure 9c,e along the *Z*-axis. Two types of defects, denominated Defects 1 and 2, were evidenced; they are highlighted in blue in Figure 9. Defect 1 (Figure 9b,c) has a similar origin to the one seen on the raw sample (Figure 7), meaning that a crack was caused by a NiO grain present at the interface between the electrolyte and the AFL. Defect 2 on (Figure 9d,e) has a different origin; it corresponds to a lack of electrolyte matter leading to a hole of ~6 µm in diameter. Nothing was found in the underlying layers that could explain the hole. Therefore, it is assumed that it comes from a growth anomaly produced during reactive magnetron sputtering. The defect arises as a result of the growth anomaly. This anomaly is removed during the annealing treatment leading to the formation of the hole.

**Figure 10:**
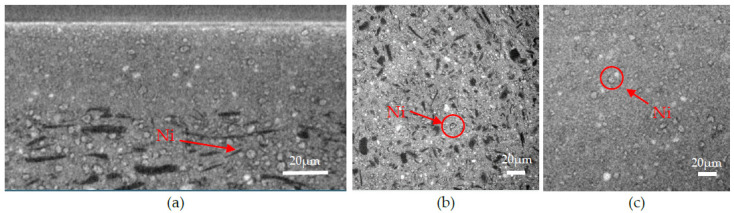
X-ray tomography images of the reduced sample: (**a**) cross-section along the *X*-axis; (**b**) cross-section of the anode along the *Y*-axis; (**c**) cross-section of the AFL along the *Y*-axis.

Finally, a reduced cell was observed. Figure 10 represents X-ray tomography images of the reduced sample. The cross-section (Figure 10a) of the sample still exhibits three distinct layers. However, as seen in the images, the microstructure has changed. The anode is still porous, and the AFL remained dense, but the NiO grains were reduced in Ni, leading to the formation of a porous shell surrounding the particles. The Ni grains are more distinguishable on the images because they are now brighter and surrounded by a pore shell, as clearly exhibited by the circles surrounding the Ni grain in Figure 10. Their smaller size can be explained by material withdrawal due to the reduction. 

**Figure 11:**
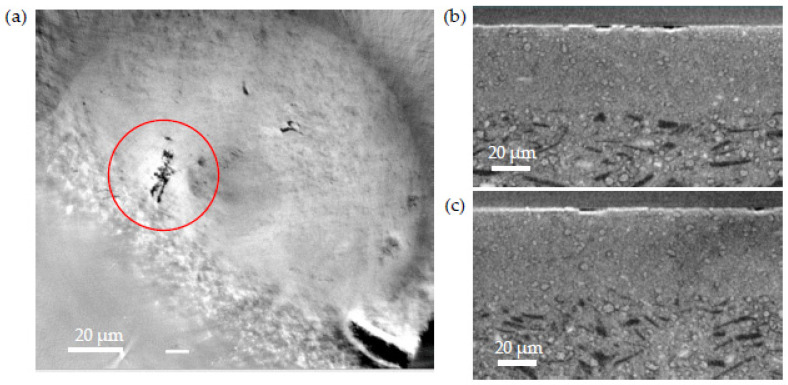
X-ray tomography images of a defect in the electrolyte of the reduced sample: (**a**) image from the top (*Y*-axis) of the defect in the electrolyte; (**b**) cross-section where the defect is along the *X*-axis; (**c**) cross-section where the defect is along the *Z*-axis.

A defect was also seen on the reduced sample, as seen in Figure 11. There is an important crack on the surface of the electrolyte. The two cross-section images show that there is nothing underneath the crack that could have caused it.

The matter withdrawal due to the reduction, combined with the TEC effect (Ni TEC being higher than NiO), leads to a strong localized reduction of the volume. This causes the collapse of the electrolyte layer in certain areas. The crack formation results from the good adhesion between the AFL and the electrolyte that remained strong during the reduction. The presence of coarse NiO grains enhanced the localized aspect of the volume reduction in some places, increasing the irregularities in the electrolyte. 

The crack is then due to the reduction of nickel oxide. The reduction leads to the contraction of the anode volume. To compensate for the compressive force exerted in the anode and the AFL, a tensile force results in the electrolyte layer leading to the electrolyte rupture. The reduction procedure has to be as smooth as possible in order to minimize the stress in the cell, but also the conclusion is the same as the raw and annealed sample, meaning that the powder has to be more homogenized during the shaping process by improving the grinding.

## 4. Conclusions

In this paper, raw, annealed, and reduced NiO-BZY20 half-cells were successively studied by X-ray micro-computed tomography (µCT). 

These studies show:

- (i) The right microstructure of the three layers composing the half-cell: a dense electrolyte, a dense anode functional layer (AFL), and a porous anode.
- (ii) A defect similar to a lack of electrolyte matter with no underlying cause, meaning that it comes from the electrolyte deposition by DC magnetron sputtering. This defect, caused by a growth anomaly, is removed during the annealing treatment leading to the formation of a hole.
- (iii) The second type of defect is due to the presence of an unusually coarse NiO grain in the AFL. Because of the difference in thermal expansion coefficient (TEC) between NiO and BZY20, the stress caused during the expansion and shrinkage of the cell during the electrolyte deposition lead to crack formation in the electrolyte.
- (iv) Finally, the last type of defect is caused by the NiO reduction. The matter withdrawal, enhanced by the presence of unusually large Ni grains, leads to localized volume reduction, causing mechanical stress in the electrolyte.

Improving the homogeneity of the starting powders would be a solution. Though other characterization techniques are possible, µCT has proved here the efficiency of tracking defects and analyzing degradation phenomena by being able to travel through the inside of the sample. Thus, the study of the origin of any defect is possible, which allows a better way to anticipate them.

## Figures and Tables

**Table 1:** Sputtering deposition parameters of Ba and Zr_0.8_Y_0.2_ targets.

Target	Power (W)	Tension (V)	Intensity (A)	Time Off (µs)	Frequency (kHz)
Ba	85	161	0.56	2	150
Zr_0.8_Y_0.2_	80	2 [30]	0.33	4	50

## Data Availability

Not applicable.
